# Comparative Analysis of MicroRNA Expression among Benign and Malignant Tongue Tissue and Plasma of Patients with Tongue Cancer

**DOI:** 10.3389/fonc.2017.00191

**Published:** 2017-08-29

**Authors:** Guilherme Rabinowits, Michaela Bowden, Ludmila M. Flores, Sigitas Verselis, Victoria Vergara, Vickie Y. Jo, Nicole Chau, Jochen Lorch, Peter S. Hammerman, Tom Thomas, Laura A. Goguen, Donald Annino, Jonathan D. Schoenfeld, Danielle N. Margalit, Roy B. Tishler, Robert I. Haddad

**Affiliations:** ^1^Medical Oncology Department, Dana-Farber Cancer Institute, Brigham and Women’s Hospital, Boston, MA, United States; ^2^Internal Medicine Department, Brigham and Women’s Hospital, Boston, MA, United States; ^3^Dana-Farber Cancer Institute, Molecular Diagnostics Laboratory, Boston, MA, United States; ^4^Anatomic Pathology Department, Brigham and Women’s Hospital, Boston, MA, United States; ^5^Radiation Oncology Department, Dana-Farber, Brigham and Women’s Hospital, Boston, MA, United States; ^6^Brigham and Women’s Hospital, Otolaryngology Division, Boston, MA, United States

**Keywords:** Exosomes, miRNA, head and neck cancer, biomarker

## Abstract

**Background:**

Identification of a microRNA (miRNA) pattern to be used as a biomarker for HNSCC is challenging given the heterogeneity of the disease and different methodologies used. To better define the field, we performed a prospective analysis of blood, tumor, and paired benign tissues in tongue squamous cell carcinoma (SCC) patients.

**Methods:**

Plasma samples were collected prior to surgery, and paired tumor and benign tissue blocks were collected from tongue cancer resections. Circulating free and exosomal miRNA, and paired tumor and benign tissues miRNA were analyzed. TaqMan-based miRNA arrays were used to quantitate the expression of 747 human miRNAs. The comparative Ct method assessed the miRNA profile results, and Student’s *t*-test determined statistical significance between tumor and benign samples.

**Results:**

Sixteen of 359 miRNAs detected were differentially expressed between paired tumor and benign tissue. Nine were upregulated, and seven downregulated in tumor tissue. All nine upregulated and six of seven downregulated tumor miRNAs were expressed in circulating exosomes. In contrast, eight of nine upregulated and four of seven downregulated tumor miRNAs were circulating free in the plasma.

**Conclusion:**

An aberrantly expressed pattern of miRNA was identified in both tumor and plasma of patients with tongue SCC, suggesting this may be a biomarker for SCC of the oral tongue. Circulating exosomes appear to be a more reliable method for evaluation of circulating tumor-miRNA expression. Further studies with a larger cohort of patients and serial blood samples are needed to validate our findings.

## Introduction

Head and neck cancer is the sixth most common cancer worldwide with squamous cell carcinoma (SCC) representing over 90% of all histologies (1). Although cure can be achieved in over 80% of those who present with early stage disease, the majority of patients are diagnosed with locally advanced disease, where 5-year overall survival has plateaued around 50% over the last few decades (1). In an effort to improve cure rates, identification of a biomarker that detects cancer at an earlier stage would be a useful screening/diagnostic tool. Thus far, no such diagnostic tool exists.

MicroRNAs (miRNAs) are small (19–25 nucleotides) non-coding RNA molecules that regulate gene expression through complementary binding to a part of their target messenger RNA sequence, degrading it or inhibiting its translation (2). Thousands of miRNAs have been reported to date, and it has been estimated that approximately 30% of all genes are regulated by at least one miRNA (3). Mutation or dysregulation in the expression of miRNA results in a gain or loss of its function, leading to downregulation or upregulation of the target protein, and functioning as oncogenes or tumor suppressor genes (4).

MicroRNAs have been extensively studied over the last few years as potential biomarkers for screening and diagnosis of cancer. Multiple studies have analyzed the miRNA expression of head and neck cancers in an attempt to identify those with diagnostic, predictive and prognostic information (5–7). Results and interpretation of these studies have been complicated by the heterogeneous group of patients, the technique utilized, and the tissue analyzed (cell lines, tumor tissue, and blood). Furthermore, lack of comparison to age, sex, matched control, the different risk factors (HPV, tobacco, and alcohol) and how they affect miRNA expression, add to the complexity of interpreting these results.

In an attempt to better define the field, we analyzed the miRNA expression of plasma, tumor and matched benign tissue of oral tongue SCC patients.

## Materials and Methods

### Patient and Tumor Characteristics

Newly diagnosed head and neck SCC patients, stages I–IV, naïve of treatment, were eligible to participate in this study. For homogeneity, only patients with oral tongue SCC undergoing surgery were analyzed here. Plasma samples of newly diagnosed tongue SCC patients were collected in EDTA tubes prior to surgery for extraction and analyses of circulating free and exosomal miRNA. Tumor and matched benign formalin-fixed in paraffin-embedded (FFPE) tissue blocks were selected from surgical resection specimens for miRNA extraction and analyses. All tumors were located in the oral tongue (anterior two-thirds) and showed morphologic features of conventional (i.e., keratinizing) SCC. Patients younger than 18 years old or with a history of metachronous or synchronous cancers were excluded. Clinical and tumor characteristics collected included age, sex, site and stage of disease, and alcohol and tobacco history. Tobacco users were defined as active, former, or never smokers. This study was approved by our institutional review board, and all patients provided written informed consent prior to tissue collection (protocol number 09-472).

### Isolation of Total RNA from Paraffin-Embedded Tissue, Plasma, and Exosomes and RNA Quantitation

H&E stains were prepared on 4 µm sections from the FFPE tissue blocks. Pathology review was undertaken to identify tumor and benign regions of interest (ROIs). Coring tools of 0.6 mm diameter were used to punch the ROI for subsequent RNA isolation. We implemented the Qiagen AllPrep DNA/RNA FFPE Kit (Qiagen, USA—cat# 80234) following the manual’s instructions for RNA extraction. TRIzol Reagent (Life Technologies, Inc., USA—cat# 15596-026) was utilized in the isolation of RNA from the matched plasma specimens, as per the manufacturer’s instructions. Exosomes were isolated with the ExoQuick serum exosome precipitation solution (Systems Biosciences, Inc., USA—cat# EXOQ5A-1) with the addition of pacific hemostasis thromboplastin D to remove coagulating material, as per the manufacturer’s instructions. The exosome solution was then subjected to TRIzol RNA isolation, as described for plasma. RNA isolates were quantified utilizing the Quant-iT RiboGreen assay (Life Technologies, Inc., USA—cat# R11490) over the concentration range 1–100 ng/µl.

### TaqMan-Based miRNA Profiling

The TaqMan^®^ Array Human miRNA platform was chosen to quantitate the miRNA expression in the series of matched samples from patients. Prior to analysis, all the RNA samples were assessed for quality assurance and concentration using the Agilent 2100 Bioanalyzer (Agilent Technologies, USA). The human 384-well TaqMan Array microfluidic two card set v3.0 (Array A and Array B, catalog no. 4444913, Life Technologies, Inc., USA) quantitates the expression of 747 unique and mature human miRNAs based on miRBase v20 (8). We determined that 60 ng of total RNA was the optimal input per sample per TaqMan array using the standard manufacturer’s recommended protocol. The plasma and exosomal samples produced much lower yields with RNA concentrations at about 1 ng/µl each. For these samples, approximately 3 ng of input RNA was used per RT reaction using the manufacturer’s protocol. Subsequently, a modified limited RNA protocol was used for the low RNA input samples. Twice the amount of cDNA product was added per preamplification reaction with thermal cycling at 95°C/10 min, 55°C/2 min, 72°C/2 min then 16 cycles of 95°C/15 s, 60°C/4 min followed by 99.9°C/10 min and 4°C on hold. The preamplified product was added undiluted to the final qPCR solution (1/100 of the total volume) and loaded on the TaqMan arrays. Standard recommended thermal cycling conditions were used for all the microfluidic cards to perform real-time PCR on a 7900HT Real-Time PCR system (Life Technologies, Inc., USA).

### Statistical Analysis

The comparative Cq method was utilized to assess the results of the miRNA profiling experiment. Initially we focused on determining the miRNAs that were differentially expressed between the tumors and the matched benign tissue. We filtered the data to exclude miRNAs that were expressed in less than 50% of the samples. If both the tumor and benign tissue had an average miRNA expression level >35 Cq units, the miRNAs were also excluded from the subsequent analysis. We calculated the mean miRNA expression for all miRNAs for a given sample and utilized this global mean value to determine the ΔCq value (9). We calculated log_2_ of the fold change to assess the differential expression between the tumor and benign ROIs. We then conducted Student’s *t*-test to determine statistical significance between the tumor and benign sample groups. *p*-Values < 0.05 were considered statistically significant. We calculated adjusted *p*-values based on the Benjamini–Hochberg test to stringently identify statistically significant differences between the tumor and benign tissues. Adjusted *p*-values < 0.05 were considered statistically significant.

## Results

Five patients with AJCC Stage II–IVa oral tongue conventional SCC were analyzed (Table 1). Sixteen of 359 miRNAs detected were differentially expressed between tumor and matched benign tissue (adjusted *p* < 0.05): 9 were upregulated (hsa-miR-19a; hsa-miR-512-3p; hsa-miR-27b; hsa-miR-20a; hsa-miR-28-3p; hsa-miR-200c; hsa-miR-151-3p; hsa-miR-223; hsa-miR-20b), and 7 downregulated (hsa-miR-22; hsa-miR-516-3p; hsa-miR-370; hsa-miR-139-5p; hsa-let-7e; hsa-miR-145-3p; hsa-miR-30c) in tumor tissue in comparison to matched benign tissue (Table 2). All nine upregulated tumor-tissue miRNAs were expressed in both tumor and plasma (both free and within exosomes), except for hsa-miR-512-3p, which was only present in tumor and within exosomes. Of the seven tumor-tissue miRNAs downregulated, four (hsa-miR-370; hsa-miR-139-5p; hsa-miR-let-7e; hsa-miR30c) were expressed in both tumor and plasma (both free and within exosomes); hsa-miR-516-3p was present in tumor only, and hsa-miR-22 and hsa-miR-145-3p were present in tumor and exosome only.

**Table 1:** Patients’ characteristics.

Patient	Site of disease	Stage	Age	Sex	Alcohol use	Tobacco use
P96	Oral tongue	T3N2b	68	Male	Active	Former
P103	Oral tongue	T2N0	68	Female	Active	Never
P114	Oral tongue	T2N1	39	Male	Active	Never
P35	Oral tongue	T3N2c	55	Female	Active	Active
P41	Oral tongue	T2N0	55	Male	Active	Active

**Table 2:** Adjusted *p*-value and log_2_ fold change of tumor versus benign tissue microRNA (miRNA) expression.

miRNA	miRBase accession number^a^	Benjamini–Hochberg adjusted *p*-value	log_2_ FC
hsa-miR-19a	MIMAT0000073	0.0036	0.32
hsa-miR-512-3p	MIMAT0002823	0.0083	0.41
hsa-miR-27b	MIMAT0000419	0.0101	0.47
hsa-miR-20a	MIMAT0000075	0.0111	0.31
hsa-miR-28-3p	MIMAT0004502	0.0135	0.40
hsa-miR-200c	MIMAT0000617	0.0138	0.29
hsa-miR-151-3p	MIMAT0000757	0.0176	0.31
hsa-miR-223	MIMAT0000280	0.0355	0.28
hsa-miR-20b	MIMAT0001413	0.0395	0.34
hsa-miR-22	MIMAT0000077	0.0027	−0.35
hsa-miR-516-3p	MIMAT0006778-MIMAT0002860	0.0096	−0.37
hsa-miR-370	MIMAT0000722	0.0111	−0.34
hsa-miR-139-5p	MIMAT0000250	0.0120	−0.37
hsa-let-7e	MIMAT0000066	0.0120	−0.42
hsa-miR-145-3p	MIMAT0004601	0.0300	−0.34
hsa-miR-30c	MIMAT0000244	0.0347	−0.35

*^a^Based on miRBase v21*.

## Discussion

To the best of our knowledge, this is the first study comparing the expression pattern of miRNA in tongue SCC, matched benign tissue and plasma (both free and within exosomes) of the same patients in an attempt to identify a signature pattern of miRNAs that would serve as a “liquid biopsy” for this patient population. All nine upregulated and six of the seven downregulated miRNAs in tumor compared to matched benign tissue were present in the circulating exosomes. Tumor cells may utilize exosomes to transport oncoMirs to other sites, leading to uncontrolled proliferation of cells away from the primary site (metastasis). Similarly, tumor cells may remain in a proliferative state by eliminating tumor suppressor-miRNAs from their environment. Indeed, eight of the upregulated and five of the downregulated miRNAs have been previously demonstrated to also be overexpressed and suppressed in other malignancies, reinforcing their role as oncogenes and tumor suppressor genes, respectively (Table 3).

**Table 3:** MicroRNAs (miRNAs) that pass stringent criteria for significance and fold change between tumor cells and matched benign tissue with its expression sites and whether previous association with cancer has been reported.

miRNA	Expression	Cancer association (Y/N)	Upregulated (Y/N)
hsa-miR-19a	Common	Y	Y (10)
hsa-miR-512-3p	T and E only	Y	N (11)
hsa-miR-27b	Common	Y	Y (12) and N (13)
hsa-miR-20a	Common	Y	Y (14)
hsa-miR-28-3p	Common	Y	Y (15)
hsa-miR-200c	Common	Y	Y (16) and N (17)
hsa-miR-151-3p	Common	Y	Y (18)
hsa-miR-223	Common	Y	Y (19) and N (15)
hsa-miR-20b	Common	Y	Y (20)
hsa-miR-22	T and E only	Y	Y (19, 21) and N (22)
hsa-miR-516-3p	T only	N	N/A
hsa-miR-370	Common	Y	Y (23)
hsa-miR-139-5p	Common	Y	N (24)
hsa-let-7e	Common	Y	N (25)
hsa-miR-145-3p	T and E only	Y	N (26)
hsa-miR-30c	Common	Y	N (27)

*T, tumor; E, exosome*.

*Common: tumor, exosome, and plasma. Purple: upregulated miRNAs. Green: downregulated miRNAs*.

Wong et al. performed a study comparing the miRNA expression pattern of tongue cancer cells and their paired normal cells in 4 patients with tongue cancer and nodal metastasis (7). Of the 156 miRNAs tested, 24 were at least 3-fold upregulated, and 13 downregulated in tumor cells in comparison to the normal cells. miRNA-184 was 59-fold higher in tumor cells in comparison to control cells, and when tested in the plasma was detected in 24 of 30 (80%) cancer patients in comparison to 5 of 38 (13%) normal individuals (7). Zhou et al. identified 25 differentially expressed miRNAs (21 overexpressed and 4 underexpressed; fold change >2; *p* < 0.01) between 15 tongue SCC patients and 3 normal controls. miR-424, miR-542-3p, and miR-454 were the most upregulated, and miR-494, miR-490-5p, and miR-486-5p were the most downregulated miRNAs in tumor in comparison to the normal tissue (28).

Boldrup et al. analyzed the expression of three miRNAs in different oral cavity subsites and highlighted the importance of taking the subsite of tumors into consideration when analyzing oral cavity SCC (29). In their study, miRNA-21 was significantly upregulated, and miRNA-125b and miRNA-203 were significantly downregulated in tongue SCC compared with clinically normal tissue adjacent to the tumors, and that pattern differed between the other oral cavity subsites analyzed (29). In a different study, Boldrup et al. evaluated the expression of miRNA-424 in tongue SCC, its adjacent clinically normal tongue and in tongue tissue of healthy individuals (30). Blood samples of some of the patients prior to surgery and of healthy individuals were also collected. miRNA-424 expression was highest in tongue SCC, followed by normal tissue of healthy individuals, and lowest in clinically normal tissue adjacent to the tumor. The authors suggested that this may have been due to the lack of normal regulation in tumor tissue. In addition, this miRNA was variably detected in plasma at very low levels with no significant difference between cancer patients and healthy individuals, suggesting that circulating miRNA-424 was not an ideal biomarker for early tongue cancer detection.

Currently, there has not been a consensus among different studies of the differentially expressed miRNAs between tumor and matched normal tissues. The small number of patients, different techniques utilized, including the miRNA platform tested, may explain some of those differences. In addition, it is unclear whether and how patient’s characteristics like sex, comorbidities, including immune status, social habits and age affect the miRNA expression pattern. Furthermore, overexpression (or upregulation) and underexpression (or downregulation) of a miRNA do not necessarily mean that miRNA function as an oncogene or tumor suppressor gene, respectively.

Arroyo et al. demonstrated that the large majority of circulating miRNAs travel in a non-membrane-bound form consistent with a ribonucleoprotein complex, while the minority travels within microvesicles and exosomes (31). Exosomes are 50–90 nm membrane-bound vesicles arising from multivesicular bodies and released from cells to the circulation by exocytosis (32). It is thought to be one the mechanisms of cell to cell communication. Whether traveling within vesicles or associated with the Argonaute2 (the effector component of the miRNA-induced silencing complex), circulating miRNAs are protected against the activity of the RNAses in the blood. Our study revealed that all upregulated, and six of the seven (86%) downregulated tumor-tissue miRNAs were seen within the circulating exosomes, suggesting that it may be a better method of detection of tumor-related miRNAs than those circulating vesicle-free in the plasma. If the minority of circulating miRNAs is within exosomes, and cancer cells are known to significantly increase the number of circulating exosomes, one may hypothesize that the majority of exosomal miRNA may indeed be tumor related.

Our study was limited by the small sample size, lack of a matched, cancer-free control group and serial circulating miRNA samples post cancer treatment.

## Conclusion

We identified an aberrantly expressed pattern of miRNAs in both tumor and plasma of patients with tongue SCC relative to matched benign tissue. In addition, the exosomal-miRNA expression pattern matched tumor miRNA more reliably than circulating free miRNA, suggesting analysis of exosomes may be a better method for prediction of tumor-miRNA expression. Whether this exosomal-miRNA expression pattern can be used as a surrogate marker for tongue SCC is unclear at this time. Further studies with a larger cohort of patients are needed to validate our findings, and serial plasma samples will be required to determine whether this method has a role to evaluate treatment response/early recurrences.

## Ethics Statement

This study was approved by Dana-Farber/Harvard Cancer Center Institutional Review Board. All patients provided written informed consent prior to tissue collection.

## Author Contributions

All coauthors reviewed, edited, and approved the final version of the manuscript. In addition, GR collected the data, analyzed and interpreted the results, and wrote the manuscript; MB, LF, and SV prepared the samples, analyzed and interpreted the results, and helped with the preparation of the manuscript. TT, LG, DA, JL, RH, and NC collected the specimens. VJ interpreted the pathology.

## Conflict of Interest Statement

None except for GR (research support to institution: EMD Serono, Exelixis, and Millennium; scientific advisory board and consulting: EMD Serono), JL (research support to institution: Novartis and Millennium), and RH (research support to institution: Merck, Bristol-Myers Squibb, Celgene, AstraZeneca, and VentiRx; consulting: Merck, Bristol-Myers Squibb, Eisai, Pfizer, and Bayer).

## References

[1] Lam L Logan RM Luke C Rees GL. Retrospective study of survival and treatment pattern in a cohort of patients with oral and oropharyngeal tongue cancers from 1987 to 2004. Oral Oncol (2007) 43:150–8. 10.1016/j.oraloncology.2005.12.030 16807069

[2] Bartel DP. MicroRNAs: target recognition and regulatory functions. Cell (2009) 136:215–33. 10.1016/j.cell.2009.01.002 19167326 PMC3794896

[3] Lewis BP Burge CB Bartel DP. Conserved seed pairing, often flanked by adenosines, indicates that thousands of human genes are microRNA targets. Cell (2005) 120:15–20. 10.1016/j.cell.2004.12.035 15652477

[4] Nicoloso MS Spizzo R Shimizu M Rossi S Calin GA MicroRNAs – the micro steering wheel of tumour metastases. Nat Rev Cancer (2009) 9:293–302. 10.1038/nrc2619 19262572

[5] Shi H Chen J Li Y Li G Zhong R Du D Identification of a six microRNA signature as a novel potential prognostic biomarker in patients with head and neck squamous cell carcinoma. Oncotarget (2016) 7(16):21579–90. 10.18632/oncotarget.7781 26933913 PMC5008307

[6] Hui L Wu H Yang N Guo X Jang X. Identification of prognostic microRNA candidates for head and neck squamous cell carcinoma. Oncol Rep (2016) 35(6):3321–30. 10.3892/or.2016.4698 27035560

[7] Wong TS Liu XB Wong BY Ng RW Yuen AP Wei WI. Mature miR-184 as potential oncogenic microRNA of squamous cell carcinoma of tongue. Clin Cancer Res (2008) 14:2588–92. 10.1158/1078-0432.CCR-07-0666 18451220

[8] Chou CH Chang NW Shrestha S Hsu SD Lin YL Lee WH miRTarBase 2016: updates to the experimentally validated miRNA-target interactions database. Nucleic Acids Res (2016) 44:D239–47. 10.1093/nar/gkv1258 26590260 PMC4702890

[9] Mestdagh P Van Vlierberghe P De Weer A Muth D Westermann F Speleman F A novel and universal method for microRNA RT-qPCR data normalization. Genome Biol (2009) 10:R64. 10.1186/gb-2009-10-6-r64 19531210 PMC2718498

[10] Feng Y Liu J Kang Y He Y Liang B Yang P miR-19a acts as an oncogenic microRNA and is up-regulated in bladder cancer. J Exp Clin Cancer Res (2014) 33:67. 10.1186/PREACCEPT-9242556491295527 25107371 PMC4237814

[11] Zhu X Gao G Chu K Yang X Ren S Li Y Inhibition of RAC1-GEF DOCK3 by miR-512-3p contributes to suppression of metastasis in non-small cell lung cancer. Int J Biochem Cell Biol (2015) 61:103–14. 10.1016/j.biocel.2015.02.005 25687035

[12] Yao J Deng B Zheng L Dou L Guo Y Guo K miR-27b is upregulated in cervical carcinogenesis and promotes cell growth and invasion by regulating CDH11 and epithelial-mesenchymal transition. Oncol Rep (2015) 35(3):1645–51. 10.3892/or.2015.4500 26706910

[13] Matsuyama R Okuzaki D Okada M Oneyama C. MicroRNA-27b suppresses tumor progression by regulating ARFGEF1 and focal adhesion signaling. Cancer Sci (2016) 107:28–35. 10.1111/cas.12834 26473412 PMC4724816

[14] Li HG Zhao LH Bao XB Sun PC Zhai BP. Meta-analysis of the differentially expressed colorectal cancer-related microRNA expression profiles. Eur Rev Med Pharmacol Sci (2014) 18:2048–57. 25027346

[15] Liu SG Qin XG Zhao BS Qi B Yao WJ Wang TY Differential expression of miRNAs in esophageal cancer tissue. Oncol Lett (2013) 5:1639–42. 10.3892/ol.2013.1251 23761828 PMC3678876

[16] Han Y Chen J Zhao X Liang C Wang Y Sun L MicroRNA expression signatures of bladder cancer revealed by deep sequencing. PLoS One (2011) 6:e18286. 10.1371/journal.pone.0018286 21464941 PMC3065473

[17] Chang L Guo F Huo B Lv Y Wang Y Liu W. Expression and clinical significance of the microRNA-200 family in gastric cancer. Oncol Lett (2015) 9:2317–24. 10.3892/ol.2015.3028 26137064 PMC4467351

[18] Sun EH Zhou Q Liu KS Wei W Wang CM Liu XF Screening miRNAs related to different subtypes of breast cancer with miRNAs microarray.Eur Rev Med Pharmacol Sci (2014) 18:2783–8. 25339470

[19] Sand M Skrygan M Sand D Georgas D Gambichler T Hahn SA Comparative microarray analysis of microRNA expression profiles in primary cutaneous malignant melanoma, cutaneous malignant melanoma metastases, and benign melanocytic nevi. Cell Tissue Res (2013) 351:85–98. 10.1007/s00441-012-1514-5 23111773

[20] Ma D Zhang YY Guo YL Li ZJ Geng L. Profiling of microRNA-mRNA reveals roles of microRNAs in cervical cancer. Chin Med J (Engl) (2012) 125:4270–6. 23217399

[21] Xu C Zheng Y Lian D Ye S Yang J Zeng Z. Analysis of microRNA expression profile identifies novel biomarkers for non-small cell lung cancer. Tumori (2015) 101:104–10. 10.5301/tj.5000224 25702651

[22] Wang G Shen N Cheng L Lin J Li K. Downregulation of miR-22 acts as an unfavorable prognostic biomarker in osteosarcoma. Tumour Biol (2015) 36:7891–5. 10.1007/s13277-015-3379-1 25953260

[23] Lo SS Hung PS Chen JH Tu HF Fang WL Chen CY Overexpression of miR-370 and downregulation of its novel target TGFbeta-RII contribute to the progression of gastric carcinoma. Oncogene (2012) 31:226–37. 10.1038/onc.2011.226 21666718

[24] Sun C Sang M Li S Sun X Yang C Xi Y Hsa-miR-139-5p inhibits proliferation and causes apoptosis associated with down-regulation of c-Met. Oncotarget (2015) 6:39756–92. 10.18632/oncotarget.5476 26497851 PMC4741860

[25] Zhao JJ Yang J Lin J Yao N Zhu Y Zheng J Identification of miRNAs associated with tumorigenesis of retinoblastoma by miRNA microarray analysis. Childs Nerv Syst (2009) 25:13–20. 10.1007/s00381-008-0701-x 18818933

[26] Karatas OF Yuceturk B Suer I Yilmaz M Cansiz H Solak M Role of miR-145 in human laryngeal squamous cell carcinoma. Head Neck (2016) 38:260–6. 10.1002/hed.23890 26083661

[27] Ling XH Han ZD Xia D He HC Jiang FN Lin ZY MicroRNA-30c serves as an independent biochemical recurrence predictor and potential tumor suppressor for prostate cancer. Mol Biol Rep (2014) 41:2779–88. 10.1007/s11033-014-3132-7 24452717

[28] Zhou XL Wu JH Wang XJ Guo FJ. Integrated microRNA-mRNA analysis revealing the potential roles of microRNAs in tongue squamous cell cancer. Mol Med Rep (2015) 12:885–94. 10.3892/mmr.2015.3467 25760063 PMC4438953

[29] Boldrup L Coates PJ Wahlgren M Laurell G Nylander K. Subsite-based alterations in miR-21, miR-125b, and miR-203 in squamous cell carcinoma of the oral cavity and correlation to important target proteins. J Carcinog (2012) 11:18. 10.4103/1477-3163.104007 23230394 PMC3515918

[30] Boldrup L Coates PJ Laurell G Wilms T Fahraeus R Nylander K. Downregulation of miRNA-424: a sign of field cancerisation in clinically normal tongue adjacent to squamous cell carcinoma. Br J Cancer (2015) 112:1760–5. 10.1038/bjc.2015.150 25965165 PMC4647232

[31] Arroyo JD Chevillet JR Kroh EM Ruf IK Pritchard CC Gibson DF Argonaute2 complexes carry a population of circulating microRNAs independent of vesicles in human plasma. Proc Natl Acad Sci U S A (2011) 108:5003–8. 10.1073/pnas.1019055108 21383194 PMC3064324

[32] Fevrier B Raposo G. Exosomes: endosomal-derived vesicles shipping extracellular messages. Curr Opin Cell Biol (2004) 16:415–21. 10.1016/j.ceb.2004.06.003 15261674

